# Radiation doses in young ICU patients: a cause for concern?

**DOI:** 10.1186/cc11129

**Published:** 2012-03-20

**Authors:** RA O'Leary, C Houlihane, P McLaughlin, M Maher, D Breen

**Affiliations:** 1 Cork University Hospital, Cork, Ireland

## Introduction

The aim of this study was to quantify the radiation dose in young ICU patients to determine if it approached a clinically significant level. Ionising radiation is a well-recognised risk factor for development of cancer. The risk is dose-related and there is no lower threshold at which the dose can be considered clinically irrelevant. The availability of computed tomography (CT) scanning has led to a significant increase in exposure to ionising radiation of patients over the last decade. Children and young adults are particularly at risk. This is partly because there is a longer lifetime in which radiation effects may be manifest but also because children are up to 10 times more sensitive to radiation than adults. In view of these issues it is important to quantify the risk to young ICU patients.

## Methods

The general ICU database was examined from 1 March 2010 to 1 March 2011. The overall radiation exposure was quantified using the cumulative effective radiation dosage (CED) in millisieverts (mSv). The CED was calculated for all of the procedures performed during the stay in the ICU using average procedure-specific effective doses published by the UK National Radiation Protection Board. A cohort of patients <30 years of age were selected for subanalysis.

## Results

There were 403 patients admitted to the general ICU during the period of interest. The number of patients <30 years of age was 75 with a mean age of 19 (range 0.5 to 30 years). The mean CED was 10.84 mSv (SD = 15.08) with 10 patients receiving >30 mSv. The mean CED for patients who did not undergo CT examination was 0.063 mSv (*n *= 31, SD = 0.062). Trauma patients received a far higher dose (21.86 mSv) than either medical (3.1 mSv) or postoperative surgical (3.96 mSv) admissions.

## Conclusion

CT is a useful and necessary tool in our diagnostic and therapeutic armoury. However, our results show that young patients can potentially be exposed to significant doses of ionising radiation in an ICU setting mainly due to CT. In view of the lifetime risk of cancer to these patients we should try to minimise radiation exposure by more judicious utilisation of CT and by use of other imaging modalities.
